# *In vivo* CD8+ T Cell Dynamics in the Liver of *Plasmodium yoelii* Immunized and Infected Mice

**DOI:** 10.1371/journal.pone.0070842

**Published:** 2013-08-14

**Authors:** Mynthia Cabrera, Lecia L. Pewe, John T. Harty, Ute Frevert

**Affiliations:** 1 Department of Microbiology, Division of Medical Parasitology, New York University School of Medicine, New York, United States of America; 2 Department of Microbiology, University of Iowa, Iowa City, Iowa, United States of America; Seattle Biomedical Research Institute, University of Washington, United States of America

## Abstract

*Plasmodium falciparum* malaria remains one of the most serious health problems globally and a protective malaria vaccine is desperately needed. Vaccination with attenuated parasites elicits multiple cellular effector mechanisms that lead to *Plasmodium* liver stage elimination. While granule-mediated cytotoxicity requires contact between CD8+ effector T cells and infected hepatocytes, cytokine secretion should allow parasite killing over longer distances. To better understand the mechanism of parasite elimination *in vivo*, we monitored the dynamics of CD8+ T cells in the livers of naïve, immunized and sporozoite-infected mice by intravital microscopy. We found that immunization of BALB/c mice with attenuated *P. yoelii* 17XNL sporozoites significantly increases the velocity of CD8+ T cells patrolling the hepatic microvasculature from 2.69±0.34 μm/min in naïve mice to 5.74±0.66 μm/min, 9.26±0.92 μm/min, and 7.11±0.73 μm/min in mice immunized with irradiated, early genetically attenuated (Pyuis4-deficient), and late genetically attenuated (Pyfabb/f-deficient) parasites, respectively. Sporozoite infection of immunized mice revealed a 97% and 63% reduction in liver stage density and volume, respectively, compared to naïve controls. To examine cellular mechanisms of immunity in *situ*, naïve mice were passively immunized with hepatic or splenic CD8+ T cells. Unexpectedly, adoptive transfer rendered the motile CD8+ T cells from immunized mice immotile in the liver of *P. yoelii* infected mice. Similarly, when mice were simultaneously inoculated with viable sporozoites and CD8+ T cells, velocities 18 h later were also significantly reduced to 0.68±0.10 μm/min, 1.53±0.22 μm/min, and 1.06±0.26 μm/min for CD8+ T cells from mice immunized with irradiated wild type sporozoites, Pyfabb/f-deficient parasites, and *P. yoelii* CS_280–288_ peptide, respectively. Because immobilized CD8+ T cells are unable to make contact with infected hepatocytes, soluble mediators could potentially play a key role in parasite elimination under these experimental conditions.

## Introduction

Despite considerable accomplishments in the fight against malaria over the past years, *Plasmodium falciparum,* the deadliest of all human malaria parasites, still remains responsible for more than half a million annual deaths worldwide, predominantly in young children in Africa [1]. In the face of the inevitable development of parasite drug resistance and potential vector resistance to insecticides, a malaria vaccine that can protect the 40% of the world's population at risk of malaria infection is therefore urgently needed. Sera and immune cells of protected individuals have identified the circumsporozoite protein (CSP) as a leading vaccine candidate [2], [3]. While encouraging, the recently completed Phase 3 testing for licensure of the CSP-based vaccine RTS, S/AS01 in African infants and young children revealed reduced risk of clinical disease only in 56% of children 5–17 months of age [4] and 31% in children 6–12 weeks of age [5]. Thus, there is a need for more efficacious second-generation malaria vaccines.

The feasibility of a fully protective malaria vaccine is supported by the attenuated sporozoite model, the first malaria vaccine shown to elicit high levels of sterile immunity in humans and experimental animals following exposure to bites of irradiated *Plasmodium*-infected mosquitoes [6], [7]. Immunization with radiation-attenuated sporozoites (RAS) remains the “gold standard” for *P. falciparum* malaria vaccine development [7]–[14]. Protection can also be achieved in mice by immunization with *P. berghei* or *P. yoelii* RAS [6], [15], [16], genetically attenuated parasites (GAP) such as the *P. yoelii* GAP Pyuis3(**−**), Pyuis4(**−**), Pyfabb/f(**−**), and PyP52/P36 double knockout in the BALB/c mouse model [17]–[21] or the *P. berghei* GAP Pbuis3(**−**), Pbuis4(**−**), PbP52(**−**), and PbP36p(**−**) in the C57Bl/6 mouse model [22]–[25]. Similarly, chemically attenuated *P. berghei* sporozoites [26], viable *P. yoelii* sporozoites under drug cover [27], and a prime boost regimen consisting of CSP peptide-coated dendritic cells and recombinant *Listeria monocytogenes* can result in immunity [15], [28]. Vaccination with late liver stage-arresting GAP without progression to blood stage development appears to generate the most efficient antimalarial immunity in mice, presumably because memory T cells targeting a broader spectrum of LS antigens are induced [18], [29].

The intricate life cycle of the *Plasmodium* parasite in the mammalian host provides a unique challenge for malaria vaccine development. After transmission into the skin by the bite of an infected mosquito [30], [31], *Plasmodium* sporozoites travel to the liver, glide along the sinusoidal endothelium, leave the bloodstream by traversing Kupffer cells [32]–[34], the resident hepatic macrophages, migrate through several hepatocytes, and develop to large intracellular liver stage (LS) parasites, which eventually differentiate to tens of thousands of erythrocyte-infective merozoites. During this extensive migration phase [35], [36], CSP and other parasite antigens are continuously released from the sporozoite surface [37]–[39] and translocated into the cytosol of mammalian cells [33], [40], [41], suggesting that essentially all parenchymal and non-parenchymal liver cells are exposed these antigens. Because most non-parenchymal liver cells, in addition to dendritic cells (DC), can function as antigen presenting cells (APC) [42], [43], sporozoite antigens released into the hepatic microenvironment are likely internalized and processed by local hepatic APC, in particular Kupffer cells, and presented to immune cells patrolling the liver sinusoids. Although not formally proven, this notion is supported by the finding that vaccination with attenuated sporozoites stimulates Kupffer cells to upregulate MHC class I and produce IL-12 [44]. In agreement with the tolerogenic properties of the liver [42], [45]–[48], however, infectious sporozoites down-modulate the production of proinflammatory cytokines and block the respiratory burst in Kupffer cells thus crippling their defense and APC function [44], [49], [50].

Immunologists, vaccinologists, and parasitologists have come to appreciate the complexity of the adaptive immune response within the hepatic microenvironment [51]. CD8+ effector memory T cells play a central role in the elimination of *Plasmodium* infected hepatocytes from the liver, both in humans and in rodent malaria models [47], [52]–[55]. *In vitro* studies have shown that *P. berghei and P. yoelii* specific CD8+ T cells are capable of contact-dependent recognition of parasite antigen on the surface of infected hepatocytes and elimination of LS in the absence of cytokines such as IFN-γ or TNF-α [56]–[58]. However, the cellular interactions and molecular effector mechanisms that lead to parasite killing *in vivo*, i.e. within the complex hepatic microenvironment of an intact immune host, are still poorly understood. Increasing evidence suggests that multiple effector mechanisms are involved in cell-mediated immunity against *Plasmodium* LS (reviewed in [47], [51], [53], [59]). Cell-mediated LS elimination could occur via classical granule-mediated cytotoxicity and require CD8+ T cell extravasation and direct contact with infected hepatocytes. Alternatively, and similar to what has been described for CD4+ helper T cells in a murine model of leishmaniasis [60], *Plasmodium* LS-specific effector T cells might recognize parasite antigen on non-parenchymal APC, secrete cytokines such as IFN-γ, and kill LS over longer distances, taking advantage of the unique lymphatic fluid pathways of the liver (reviewed in [61]).

The dynamics of CD8+ effector T cells in the hepatic microenvironment have not been described to date, neither in the liver of *Plasmodium-*immunized mice nor after adoptive transfer into *Plasmodium*-infected mice. The objective of this work was to directly visualize, under dynamic *in vivo* conditions via intravital microscopy (IVM), the interactions between CD8+ effector T cells and *P. yoelii* LS in the liver of an immune host. In this first demonstration of the dynamic CD8+ T cell behavior in the liver, we show that immunization of BALB/c mice with Py-RAS, Pyuis3(**−**), Pyuis4(**−**), or Pyfabb/f(**−**) significantly increases the velocity of CD8+ T cells patrolling the sinusoids compared to naïve mice. Surprisingly, however, these motile CD8+ T cells lost their ability to migrate and remained stationary in the hepatic microvasculature of *P. yoelii* infected mice for at least three days after adoptive transfer. Because the adoptively transferred CD8+ T cells were not observed to extravasate into the hepatic parenchyma or make contact with infected hepatocytes, we propose that soluble mediators are responsible for parasite elimination from the liver under these experimental conditions.

## Materials and Methods

### Ethics statement

This study was conducted in strict accordance with the recommendations in the Guide for the Care and Use of Laboratory Animals of the National Institutes of Health. The protocol was approved by the Institutional Animal Care and Use Committee, NYU School of Medicine (Protocol number 120213-01). All surgery was performed under ketamine-xylazine-acepromazine anesthesia, and all efforts were made to minimize suffering.

### Parasites

Wild type *P. yoelii* strain 17XNL (PyXNL), *P. yoelii* 17XNL expressing GFP (PyXNL-GFP) [62], and *P. yoelii* 17XNL GAP with a deletion at *PyUIS4* [Pyuis4(**−**)] [17] or *PyFabB/F* [Pyfabb/f(**−**)] [18] were propagated in female Swiss Webster mice (Taconic Farms) and cycled in female *Anopheles stephensi* mosquitoes.

### Mice and *P. yoelii* infection

BALB/c mice (Taconic Farms), in particular the BALB/cAnNHsd strain (Harlan Laboratories) were considered best for LS IVM due to their high susceptibility to infection with *P. yoelii* sporozoites. Tie2-GFP mice, which express GFP in vascular endothelia [63] (TgN(TIE2GFP)287Sato/J, Stock 003658, Jackson Laboratory, Bar Harbor, ME), were backcrossed into BALB/c background [64]. LS development was initiated by intravenous injection of at 1–2×10^6^ freshly dissected sporozoites in mice. Sporozoites were isolated from salivary glands of infected mosquitoes by gently crushing the glands with a micropestle in a microtube (Kimble Chase, 749520–0000) with minimal RPMI medium (Invitrogen, 11835–055) with 2% fetal bovine serum (HyClone) [32], [65]. After sequential centrifugation at 700 rpm and 500 rpm, sporozoites were recovered from the supernatant fractions and resuspended to a final volume of 200 μl with RPMI.

### Immunization and challenge

To produce radiation-attenuated sporozoites (Py-RAS), freshly isolated Py17XNL sporozoites were irradiated within a gamma irradiator (MDS Nordion Gammacell 1000 Elite) to a central dose of ±12,049 cGy and a minimum dose of ±10,266 cGy. Recipient BALB/c mice were injected intravenously with 50,000 Py-RAS, with 2 subsequent booster injections of 20,000 Py-RAS each, at 15 days intervals. A second and third group of BALB/c mice were immunized with freshly isolated Pyuis4(**−**) and Pyfabb/f(**−**) GAP sporozoites using the same dosing scheme. To elicit large numbers of Py-CS_280–288_ epitope-specific CD8+ T cells, a fourth group of BALB/c mice was primed with 1×10^6^ splenic dendritic cells coated with *P. yoelii* CS_280–288_ and boosted 7 d later with 1×10^4^ – 1×10^7^ CFU recombinant *Listeria monocytogenes* expressing CS_280–288,_ as described previously [66], [67]. For *ex vivo* analysis of LS, immunized mice were challenged with 300,000 viable PyGFP-XNL sporozoites. As positive controls, naïve mice were challenged at the same time by inoculation of the same number of sporozoites from the same pool of dissected sporozoites.

### Splenic and hepatic lymphocyte isolation and adoptive transfer

For adoptive transfer, the entire population of hepatic or splenic CD8+ cells was harvested from immunized mice 2 weeks after the second booster, and suspended in 300 μl RPMI. Single cell suspensions of splenic lymphocytes were obtained by mechanically disrupting spleens and filtering through a 100 μm cell strainer (Fisherbrand, Fisher Scientific, 22-363-549). Erythrocytes were lysed with ACK Lysis Buffer (Lonza, 10-548E) for 10 min at RT, washed and resuspended in PBS pH 7.0. Total numbers of splenic and CD8+ cells (eBiosciences, 12-0081) were determined using a BD Accuri C6 flow cytometer (Becton Dickinson). For isolation of intrahepatic lymphocytes (IHL), livers from Py-RAS immunized mice were perfused using a modified perfusion method by Crispe [68]. Briefly, livers were perfused with 95% O_2_/5% CO_2_ balanced buffers to remove contaminating erythrocytes from the sinusoidal space, homogenized, digested with collagenase, and enriched via density gradient centrifugation with Optiprep Density Gradient Medium (Sigma-Aldrich). CS_280–288_ specific CD8+ T cells were prepared as described [67]. For all immunization conditions, 10^6^ CD8+ T cells were injected intravenously into anesthetized recipient mice at the designated time points.

### Anesthesia, surgery, and intravital microscopy

For intravital microscopy, mice were anesthetized with a cocktail of 50 mg/kg Ketamine HCl (Ketaset, Fort Dodge Animal Health, 0856-4403-01), 10 mg/kg Xylazine (Lloyd Laboratories, 4821), and 1.7 mg/kg Acepromazine Maleate (Butler Animal Health Supply, 003845) (KXA mix) as described [35], [64], [65]. The mouse peritoneal cavity was opened along the rib cage and the liver exposed for IVM as described [35], [64], [65] using an inverted Leica DMIRE2 microscope equipped with a temperature controlled Ludin chamber, and analyzed with a Leica TCS SP2 AOBS confocal system (40x HCX PL APO 1.25–0.75 oil lens) with the following excitation light sources: 405 nm Blue Diode laser; 488 nm Argon/ Krypton line; and HeNe laser lines at 543 nm, 594 nm and 633 nm. Periodic reinjection of KXA mix at 60–90 minute intervals allowed imaging for at least 4 h.

### Cellular *in vivo* markers

Prior to imaging, mice were intravenously or intraperitoneally inoculated with 648 nmol of the nuclear stain Hoechst 33342 (λ_ex_  = 405 nm; Invitrogen). To visualize hepatocytes, mice received 18.4 nmol MitoTracker Deep Red (λ_ex_  = 633 nm; Invitrogen) or 14.9 nmol MitoTracker Green FM (λ_ex_  = 633 nm; Invitrogen) as described previously [64]. CD8+ T cells were either surface-labeled by intravenous injection of 2 μg phycoerythrin (PE)-conjugated anti-mouse CD8a (Clone 53–6.7, λ_ex_  = 488 nm; eBiosciences) or loaded after purification with CellTracker Red CMTPX (Invitrogen) or Cell Trace Calcein Red-Orange AM (λ _ex_  = 594 nm) or Cell Trace Calcein Violet AM (λ_ex_  = 405 nm) and then inoculated intravenously as described previously [64].

### *Ex vivo* analysis of LS number and size

Anesthetized mice were intravenously injected with fluorescent tracers and antibodies and sacrificed 20 min later. The liver was dissected out and immersed in ice-cold PBS pH 7.0 for preparation of 200 μm thick tissue sections (speed 0.60 mm/s and amplitude 1.0 mm) using a Leica VT 1200 S vibratome. Liver sections were kept hydrated in PBS pH 7.0, lined up on microscope slides, covered with a 22×50 coverslip (Fisherbrand No 1.5, Fisher Scientific; 12-544-D), and sealed with nail polish. The sections of fresh unfixed liver tissue were immediately systematically scanned visually. Each 40x field of view was defined as a dimension of 373×373×∼50 μm observed immediately using a Leica DMIRE2 microscope and subsequently analyzed with a Leica TCS SP2 AOBS confocal system (40x HCX PL 1.25–0.75 oil lens). A field of view with no LS parasite was still counted as part of the total number of fields of view. XYZ stacks were collected for LS parasite volume determination.

### Image processing

After acquisition with Leica LCS software, 2D, 3D, or 4D datasets were reconstructed and processed in Imaris 7.4.2 (Bitplane). LS volume was measured using isosurface reconstructions and tracks were measured by tracking ROIs as spots or isosurfaces. Volume and track statistics obtained from Imaris software were exported to Microsoft Excel and SigmaPlot 12.0 (Systat Software Inc.). The arrest coefficient is defined as the proportion of time individual CD8+ cells exhibit a velocity of less than 2 μm/min. Composite figures were generated in Adobe Photoshop (Adobe Systems Inc.).

### Statistic analyses

Velocity and arrest coefficient statistics were calculated using One-way ANOVA on Ranks. LS number and size statistics were calculated using unpaired t-tests. Errors are standard error of the mean ± SEM.

## Results

The goal of this study was to better understand the cellular dynamics associated with the elimination from the liver of *Plasmodium* LS by CD8+ effector T cells. In a first set of experiments, we generated CD8+ effector memory T cells specific for *P. yoelii* 17NXL LS by immunizing BALB/c mice with Py-RAS or GAP sporozoites using standard intravenous immunization schemes [69]. Using our recently developed novel imaging techniques for the hepatic microvasculature [64], we monitored the behavior of these CD8+ T cells in the sinusoidal microvasculature of immunized mice and, for comparison, naïve mice by IVM. CD8+ T cells were visualized *in vivo* either by loading with CellTracker or CellTrace dyes prior to adoptive transfer or by intravenous inoculation of fluorochrome-conjugated anti-mouse CD8a antibody after transfer, labeling conditions that do not interfere with the ability of cytotoxic T cells to crawl or to kill antigen-presenting target cells *in vivo* or *in vitro* (Cabrera, Movila, Nacer, and Frevert, unpublished data) [70]–[72].

### CD8+ T cell effector function

**Figure 1:**
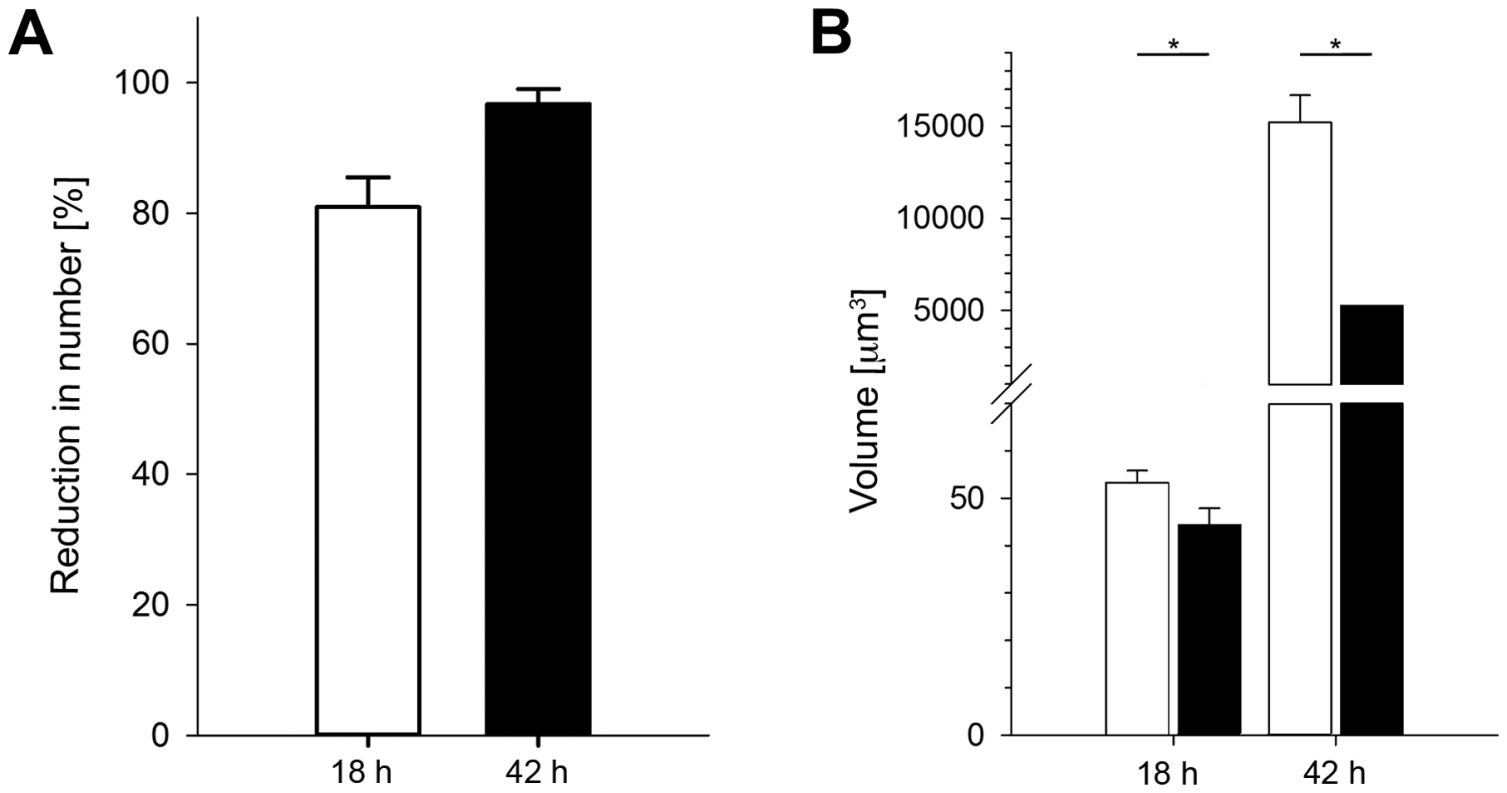
Inhibition of LS development after infection of immunized mice. Naïve or Pyfabb/f(**−**) immunized BALB/c mice were infected with 3×10^5^ PyXNL-GFP sporozoites. Immunized mice were infected 2 weeks after the second booster. At 18 h or 42 h post-infection, livers were removed and LS parasites were quantified in fresh unfixed liver tissue. Using vibratome sections of known thickness and a region of interest of a defined size, the number of LS was counted and normalized to a volume of 1.3 cm^3^ (approximate liver volume of adult mice). Panels (**A**) and (**B**) show the reduction in LS number and volume, respectively, in Pyfabb/f(**−**) immunized mice compared to naïve mice. *  =  p<0.05.

Pyfabb/f(**−**) sporozoite immunized BALB/c mice have been shown to be develop CD8+ T cell mediated protective immunity [18]. To assess the effector function of the CD8+ T cells within the immunized mice, naïve or Pyfabb/f(**−**) immunized mice were challenged with 3×10^5^ viable PyXNL-GFP sporozoites and sacrificed 18 h or 42 h later. *Ex vivo* measurement at 18 h and 42 h after challenge of LS number to measure parasite density in the liver tissue and LS size to detect changes in growth rate revealed a reduction of 81% and 97%, respectively, in PyXNL-GFP LS in Pyfabb/f(**−**) immunized mice relative to naive control mice (**Fig.** **1A**). In addition, the volume of the few remaining LS was significantly reduced by 63% (p<0.05) at 42 h post infection, from 14,177±1556 μm^3^ in the control mice to 5306±2517 μm^3^ in the immunized mice (**Fig.** **1B**). In combination, the 97% and 63% reduction in LS density and volume, respectively, translates into a near-complete prevention of LS development in the immunized mice.

### CD8+ T cell motility in naïve and immunized mice

**Figure 2:**
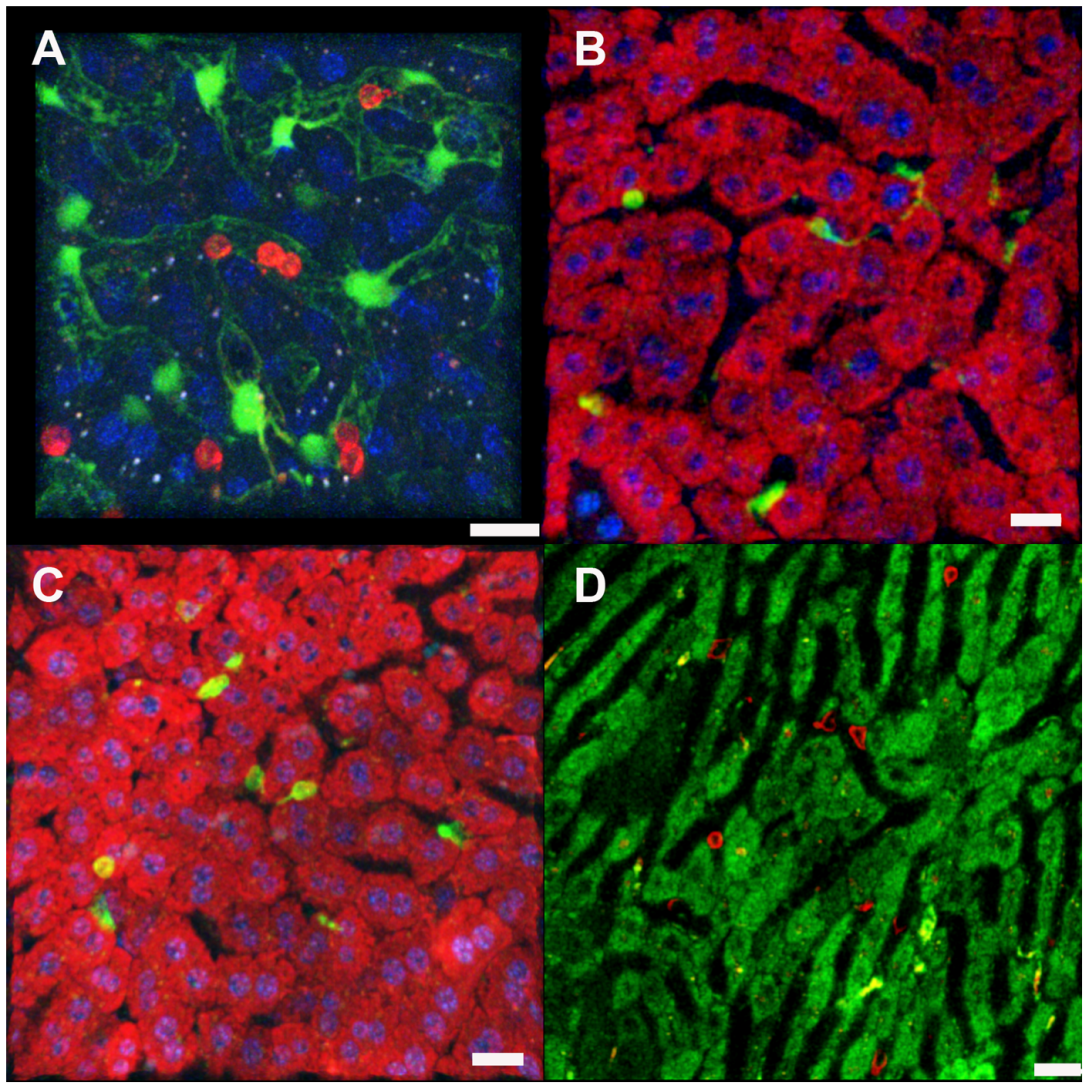
Behavior of CD8+ T cells in the liver of naïve and immunized mice. Representative IVM images of CD8+ T cells observed in naïve and *P. yoelii* immunized mouse livers. Note that in contrast to naïve mice, the T cells from immunized mice exhibit the characteristic amoeboid shape of activated effector cells and patrol the sinusoids with a leading edge and a trailing pseudopod. (**A**) 3D representation of a naïve Tie2-GFP mouse liver with fluorescent endothelia (green), anti-CD8a-PE labeled CD8+ T cells (red), and Hoechst stained nuclei (blue). (**B** and **C**) 3D projections of anti-CD8a-PE labeled CD8+ T cells (green) in the livers of mice immunized with (**B**) Pyfabb/f(**−**)or (**C**) Py-RAS. Hepatocyte mitochondria were labeled with MitoTracker (red) and nuclei were stained with Hoechst (blue). (**D**) 2D snapshot of the liver of a mouse immunized with Pyuis4(**−**) showing CD8+ T cells labeled with anti-CD8a-PE (red). Hepatocyte mitochondria labeled with MitoTracker (green). CD8+ T cells of immunized mice were monitored 2 weeks after the second booster. See **Videos S1, S2, S3, S4** for the corresponding movies. Scale bars 20 μm.

To visualize effector CD8+ T cells *in situ*, we used intravital microscopy combined with intravenous injection of a PE-conjugated anti-CD8a antibody to visualize the cells within the liver microenvironment. CD8+ T cells in naïve mice had a rounded shape and either remained stationary in the sinusoids or moved at bloodstream velocity in the livers of naïve mice (**Fig.** **2A** **, Video S1**). In contrast, CD8+ T cells from immunized mice imaged 2 weeks after the second booster immunization exhibited the characteristic amoeboid shape and behavior of activated effector T cells with a leading edge and a trailing uropod and actively patrolled the liver sinusoids of BALB/c mice immunized with Pyfabb/f(**−**) sporozoites (**Fig.** **2B** **, Video S2**), Similar observations were made in mice immunized with Py-RAS (**Fig.** **2C** **, Video S3**) or Pyuis4(**−**) (**Fig.** **2D** **, Video S4**) sporozoites, which also develop high levels of CD8+ T cell mediated immunity [73]–[75].

**Figure 3:**
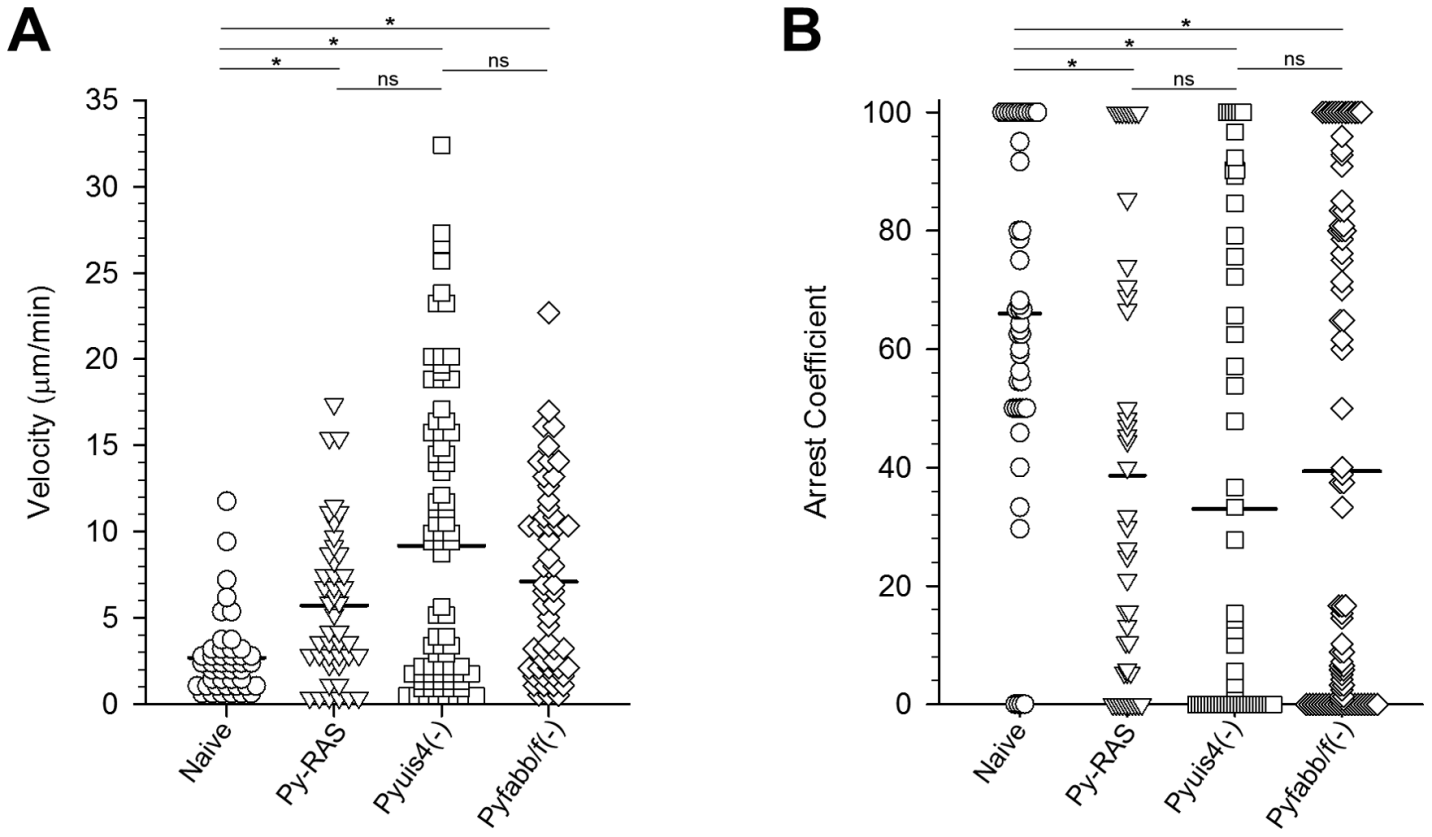
Tracking CD8+ cells in naïve and immunized mouse livers. CD8+ T cells were identified by anti-CD8a-PE labeling in the livers of naïve mice (circles) or mice immunized with Py-RAS (triangles), Pyuis4(**−**) (diamonds), or Pyfabb/f(**−**) (squares) and monitored via IVM. Velocities (**A**) and arrest coefficients (**B**) were calculated from the tracks of individual CD8+ T cell from at least 2 infected mice per group. One-way ANOVA on Ranks shows that the velocities of the CD8+ T cells from all immunized mice are significantly higher and the arrest coefficients significantly lower than those of the CD8+ T cells from naive mice (p<0.05). The same statistical analysis does not reveal any significant difference between the CD8+ T cell velocities between the groups of immunized mice (p>0.05). *  =  p<0.005, ns  =  not significant. CD8+ T cells of immunized mice were monitored at least 2 weeks after the second booster.

To quantify these IVM observations, we measured velocities of individual CD8+ T cells in the livers of naïve and immunized mice (**Fig.** **3A**). CD8+ T cells from naïve mice crawled at the slow speed of 2.69±0.34 μm/min, which has been defined as locally confined movement [76]. In contrast, velocities of CD8+ T cells from the sporozoite immunized mice measured 2 weeks after the second booster immunization were significantly higher (Py-RAS  = 5.74±0.66 μm/min, Pyuis4(**−**)  = 9.26±0.92 μm/min, and Pyfabb/f(**−**)  = 7.11±0.73 μm/min; p<0.05). The CD8+ T cell velocities amongst the groups of mice immunized with Py-RAS, Pyuis4(**−**), and Pyfabb/f(**−**) were not significantly different (p>0.05). Calculation of the corresponding arrest coefficients (**Fig.** **3B**) revealed that the majority of the CD8+ T cells (66%) in naïve mice moved at a speed of less than 2 μm/min. In contrast, the arrest coefficients of the CD8+ T cell populations in mice immunized with Py-RAS, Pyuis4(**−**), or Pyfabb/f(**−**) sporozoites were significantly lower (p<0.05), with only 39%, 33%, and 39%, respectively, of the cells moving at less than 2 μm/min. Thus, the velocity of CD8+ T cells monitoring the liver sinusoids in immunized mice was significantly increased compared to naïve mice. Considering that effector memory T cells represent the majority of liver resident CD8+ T cells one week after challenge [25], the increased velocity likely reflects an antigen-specific effect.

### Behavior of CD8+ T cells after adoptive transfer into *Plasmodium* infected mice

Accumulating evidence suggests that immunization with attenuated sporozoites generates a reservoir of hepatic CD8+ T cells, which is maintained by Kupffer cell and DC derived IL-15 in the presence of a depot of parasite LS antigens, and that resident CD44^hi^CD45RB^hi^CD122^hi^CD62L^lo/hi^ CD8+ central memory T cells are required for the proliferation of IFN-γ producing CD44^hi^CD45RB^lo^CD122^lo^CD62L^lo^ effector memory T cells capable of conferring protection against reinfection [55], [77]–[79]. Based on these findings, we performed all preliminary experiments with purified IHL [68]. To do this, naïve BALB/c mice were intravenously inoculated with 1–2×10^6^ PyXNL-GFP sporozoites. The mice were surgically prepared for IVM and a well-immobilized area of the exposed liver lobe was selected for long-term observation of LS [35], [65]. The mice were then inoculated with 10^6^ fluorescently labeled CD8+ T cells, which had been isolated from the livers of Py-RAS or Pyuis4(−) sporozoite immunized mice. Initially, multiple sets of experiments were conducted in which the timing of T cell purification relative to challenge, adoptive T cell transfer relative to sporozoite inoculation, and IVM timing relative to infection and adoptive transfer were varied in an effort to optimize the conditions that favor interactions between CD8+ T cells and infected hepatocytes in the recipient mice. When the CD8+ T cells remained essentially immotile independently of the experimental conditions tested, we settled for a prime-boost regimen that mirrors an immunization scheme that protects mice against challenge with 10,000 PyXNL sporozoites [20]. CD8+ T cells were harvested from donor mice 2 weeks after the second booster and transferred into recipient mice 18 h or 42 h post-infection, followed immediately by IVM examination.

To exclude the possibility that liver perfusion or other procedures required for purification of intrahepatic lymphocytes (IHL) [68] abrogated motility, we repeated the experiments with splenic T cells. Surprisingly, the velocity of immune splenic CD8+ T cells was not significantly different (>0.05) from that of immune IHL at 18 h or 40 h after adoptive transfer into PyXNL-GFP infected mice (**Fig. S1**). Both hepatic and splenic CD8+ T cells were immotile when imaged immediately after adoptive transfer into uninfected naïve control mice (data not shown). Because no differences could be observed for the overall behavior of CD8+ T cells purified from liver versus spleen under the different infection, immunization, and IVM conditions tested, including transfer into uninfected control mice, all subsequent experiments were done with splenic CD8+ T cells.

**Figure 4:**
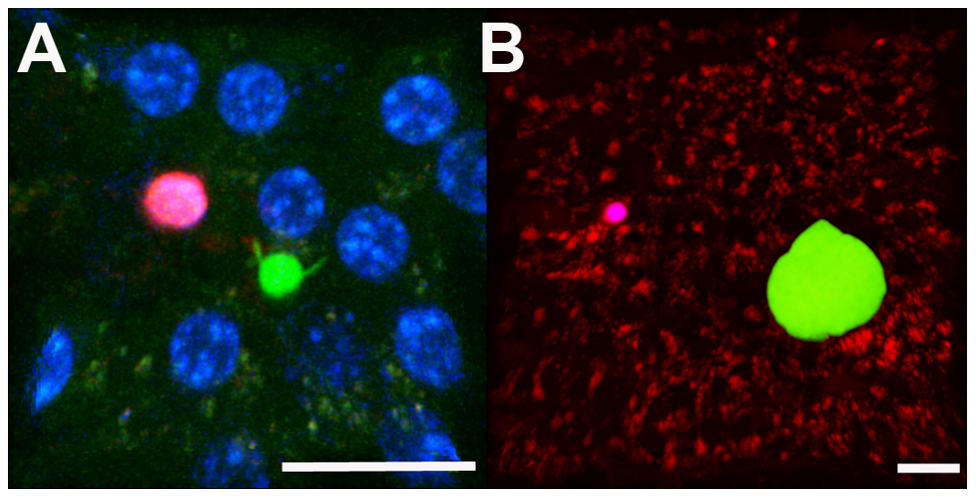
Behavior of adoptively transferred CD8+ T cells in the liver of infected mice. At least 2 weeks after the second booster, CD8+ T cells were purified from the spleens of Py-RAS immunized mice and loaded with CellTracker Red. One million CD8+ T cells were transferred into recipient mice 18 h (**A**) or 42 h (**B**) post infection with PyXNL-GFP, and immediately imaged by IVM. CD8+ T cells (red) remained immobile and failed to migrate to or make contact with hepatocytes infected with PyXNL-GFP LS (green). Hepatocyte autofluorescence is shown in green in (**A**) and red in (**B**). Nuclei were stained with Hoechst (blue) in (**A**). Scale bars 20 μm.

**Figure 5:**
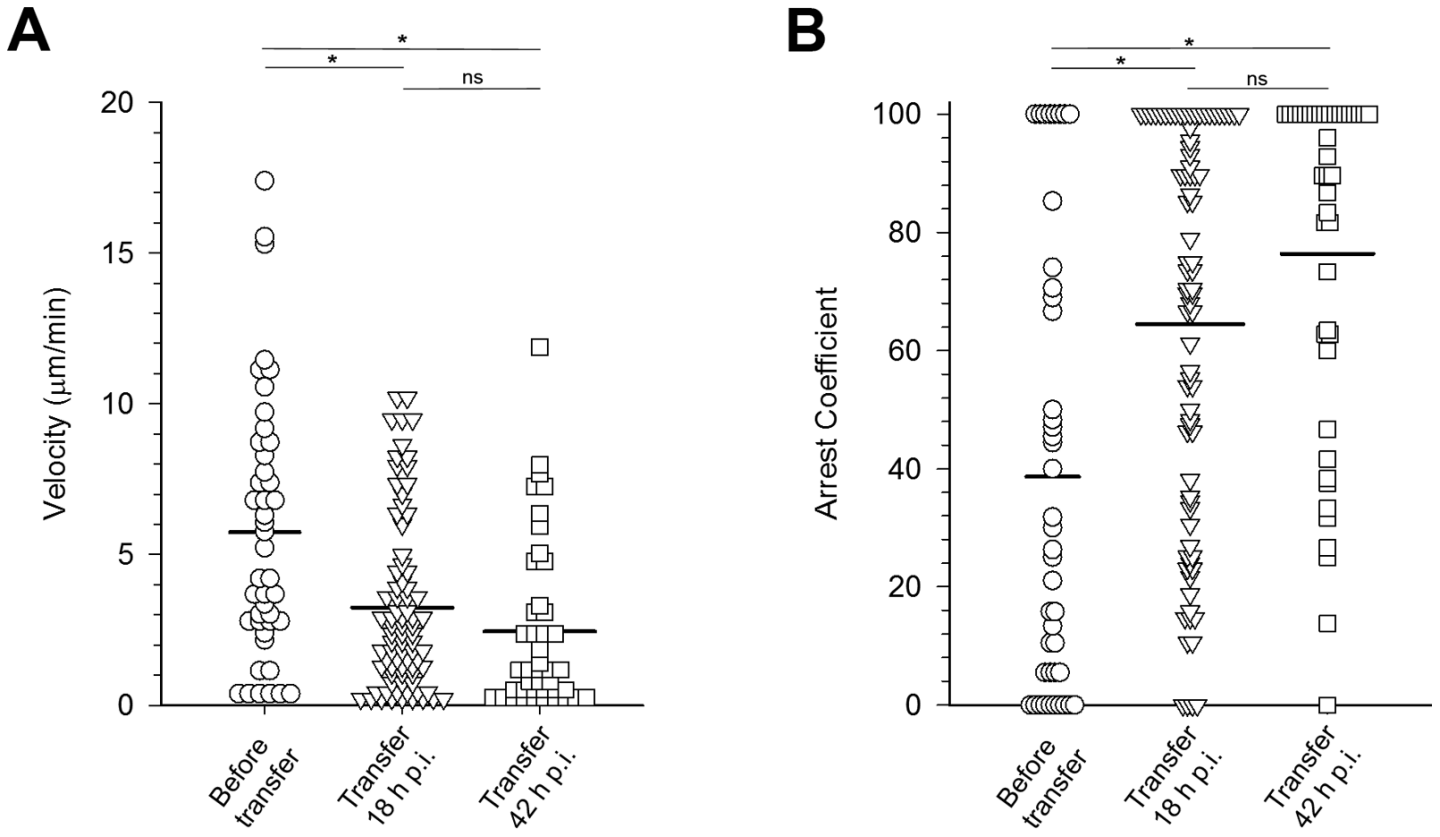
Tracking adoptively transferred Py-RAS activated CD8+ T cells in the livers of infected mice. LS were identified in a well-immobilized area of the liver and labeled CD8+ T cells in the vicinity of the LS were monitored to calculated velocities (**A**) and arrest coefficients (**B**). Py-RAS CD8+ T cells in the liver of immunized mice (circles) were used as controls. CD8+ T cells were purified 2 weeks after the second booster from the spleens of Py-RAS immunized mice and 1 million was adoptively transferred into recipient mice 18 h (triangles) or 42 h (squares) after infection with PyXNL-GFP. Based on ANOVA on Ranks, the velocity and arrest coefficients of all adoptively transferred cells differed significantly from Py-RAS in the liver of immunized mice (p<0.05). The same test showed only a significant difference in the values between the two 18 h time points. At least nine infected mice were used per experimental condition. *  =  p<0.05, ns  =  not significant.

**Figure 6:**
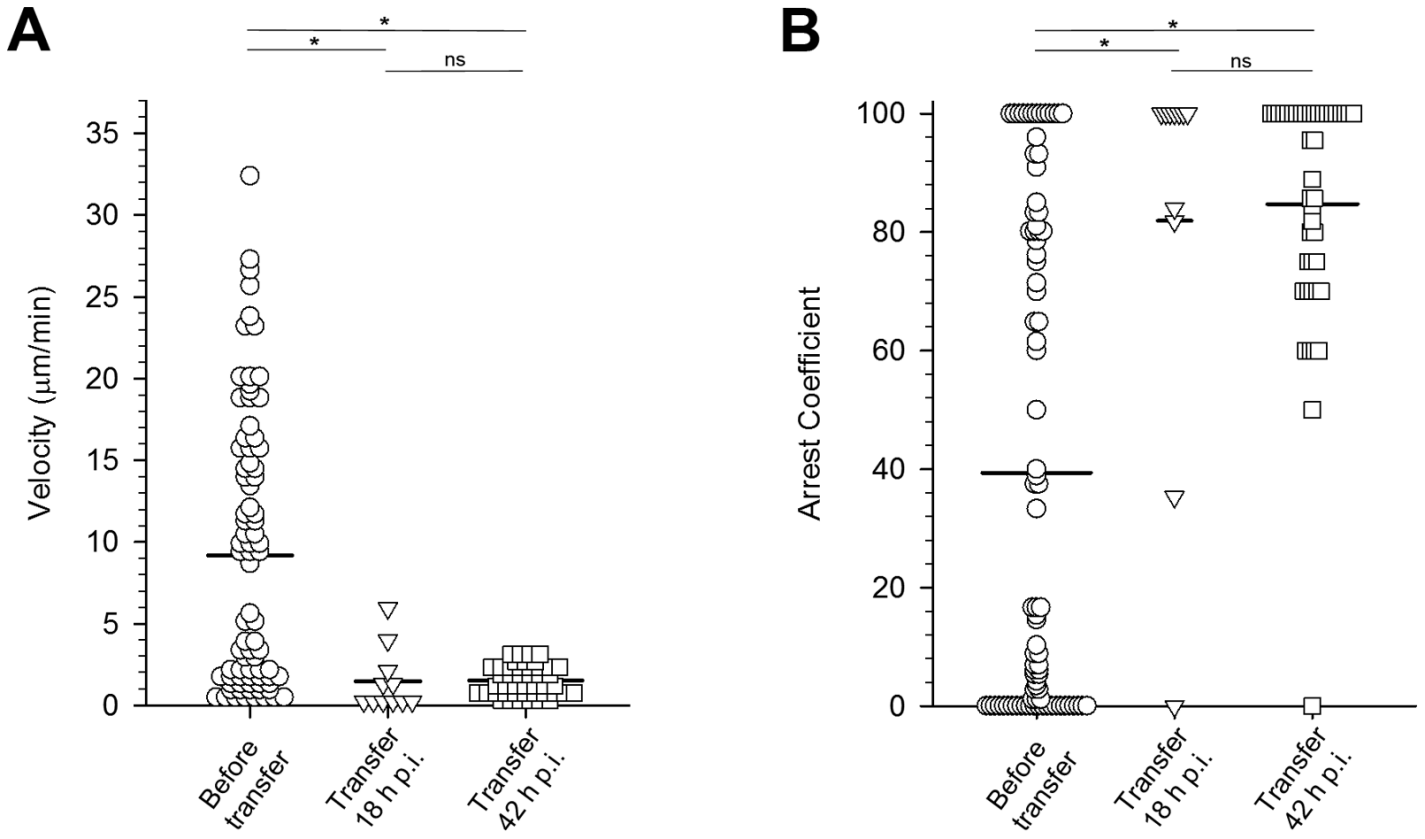
Tracking adoptively transferred Pyuis4(−) activated CD8+ T cells in the livers of infected mice. Mice were infected with 1–2 million PyXNL-GFP sporozoites. Activated CMTPX labeled CD8+ T cells were then purified 2 weeks after the second booster from the spleens of Py-RAS immunized mice and 1 million was adoptively transferred into the infected mice at 18 h (triangles) or 42 h (squares) after infection with PyXNL-GFP. Anti-mouse CD8a labeled T cells in Pyuis4(−) immunized mice (circles) were used as controls. ANOVA on Ranks show that the mean velocities (**A**) and arrest coefficients (**B**) of all adoptively transferred CD8+ T cells differ significantly from those of the CD8+ T cells in Pyuis4(−) immunized mice. At least three infected mice were used per experimental condition. *  =  p<0.05, ns  =  not significant.

At 16–18 h post-infection, young LS did not yet occupy the entire cytoplasm of the infected hepatocyte and frequently had not yet retracted their sporozoite ends (**Fig.** **4A** **, Video S5**). Only beyond 20 h after infection did the LS appear completely rounded (data not shown). When harvested from donor mice at least 2 weeks after the second booster immunization with Py-RAS and transferred (1 million) into recipient mice harboring early stage LS, splenic CD8+ T cells moved at 3.24±0.32 μm/min (**Fig.** **5A**), which is significantly slower than the CD8+ T cells observed in the Py-RAS immunized mice (5.74±0.66 μm/min; p<0.05). By 42 h post-infection, *P. yoelii* LS are almost mature and exceed the size of normal uninfected hepatocytes (**Fig.** **4B** **, Video S6**). Splenic CD8+ T cells harvested from Py-RAS immunized mice 2 weeks after the second booster immunization and adoptively transferred (1 million) into mice harboring late stage LS also moved at the significantly slower velocity of 2.44±0.43 μm/min. In the PyXNL-GFP infected BALB/c mice, the average arrest coefficients for CD8+ T cells adoptively transferred from Py-RAS immunized mice revealed significantly higher numbers of CD8+ T cells with track velocities of less than 2 μm/min (p<0.05) at 18 h (64%) and 42 h (76%) post infection when compared to the CD8+ T cell arrest coefficient in immunized mice (**Fig.** **5B**). Similarly, when compared to mean velocities of CD8+ T cells in the livers of Pyuis4(−) sporozoite immunized mice (9.18±0.94 μm/min), the mean velocities of splenic CD8+ T cells adoptively transferred into PyXNL-GFP infected mice were significantly lower when adoptive transfer was done at 18 h (1.45±0.57 μm/min) and 42 h (1.51±0.14 μm/min) post infection (**Fig.** **6A**). Again, the corresponding arrest coefficients followed the pattern observed for Py-RAS immunization: compared to the CD8+ cells in Pyuis4(−) sporozoite immunized mice (39%), 82% and 85% of the CD8+ T cells at 18 h and 40 h, respectively, had a velocity of less than 2 μm/min after adoptive transfer (p<0.05) (**Fig.** **6B**). Significantly, none of the adoptively transferred CD8+ T cells observed (N = 197) made direct contact with LS at any time least during the 4 h IVM monitoring of a total of 25 infected mice. If in rare instances CD8+ T cells were arrested in the vicinity of infected hepatocytes, they showed no evidence for a change in shape, which would precede formation of an immunological synapse indicating recognition of cognate peptide on infected hepatocytes [72].

### Simultaneous sporozoite infection and adoptive CD8+ T cell transfer

**Figure 7:**
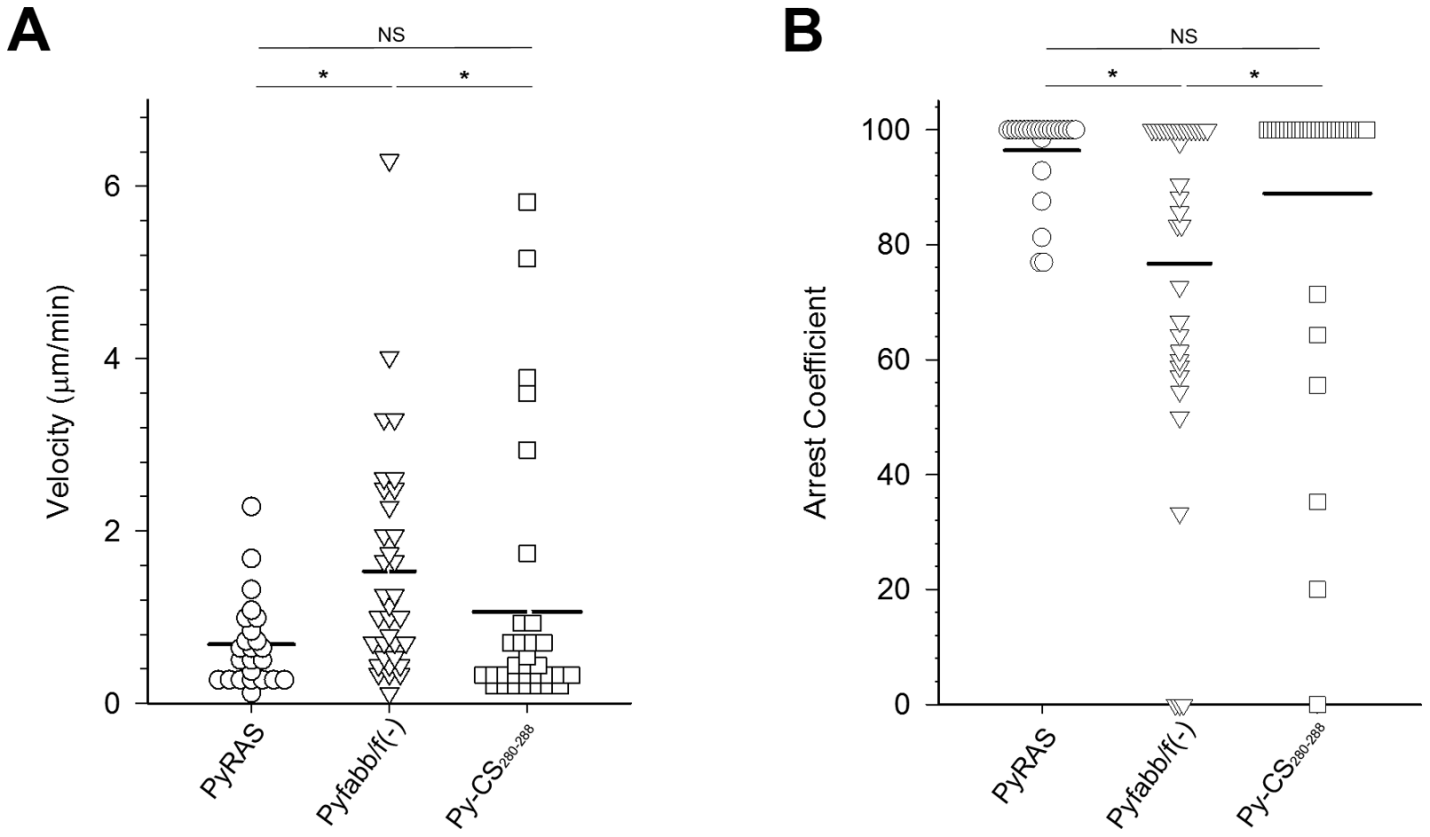
Tracking adoptively transferred Py-RAS and Pyfabb/f(−) activated CD8+ T cells in the livers of simultaneously infected mice. Mice were injected intravenously with 1–2 million PyXNL-GFP sporozoites and then immediately inoculated with 1 million labeled CD8+ T cells purified from the spleens of Py-RAS (circles), Pyfabb/f(−) (triangles), or Py-CS_280–288_ (squares) immunized mice 2 weeks after the second booster. Mean velocities (**A**) and arrest coefficients (**B**) of CD8+ T cells from mice immunized with Py-RAS versus Pyfabb/f(−) and Pyfabb/f(−) versus Py-CS_280–288_ differed significantly from each other (one-way ANOVA on Ranks). No significant difference was found for PyRAS versus Py-CS_280–288_. Adoptive transfer of CD8+ T cells into infected BALB/c mice was done immediately after PyXNL-GFP sporozoite inoculation. IVM was performed at 18 h post-infection. At least three mice were analyzed per experimental condition. *  =  p<0.05; NS  =  p>0.05.

Since RAS parasite arrest occurs at early stages of development, we addressed the possibility that CD8+ T cells recognize infected hepatocytes only at early stages of LS development. BALB/c mice were inoculated with 1–2×10^6^ PyXNL-GFP sporozoites immediately followed by adoptive transfer 10^6^ splenic CD8+ T cells harvested from Py-RAS or Pyfabb/f(−) immunized mice 2 weeks after the second booster immunization and monitored by IVM 18 h later. Compared to the CD8+ T cell velocity in the Py-RAS immunized donor mice before transfer (5.74±0.66 μm/min; **Fig.** **3A**), the average velocity of the Py-RAS CD8+ T cells after transfer into the PyXNL-infected mice was 0.68±0.10 μm/min **Fig.** **7A** **, Video S7**) and thus significantly lower (p<0.05). The corresponding arrest coefficient for CD8+ T cells after transfer was 96% (**Fig.** **7B**) compared to 39% for the immunized donor mice before transfer (**Fig.** **3B**). Similarly, the average velocity of CD8+ T cells from Pyfabb/f(−) immunized mice was 1.53±0.22 μm/min after transfer into PyXNL-infected mice (**Fig.** **7A**), which is significantly lower (p<0.05) than the value of 7.11±0.73 μm/min in the Pyfabb/f(−) immunized donor mice before transfer (see **Fig.** **3A**). Again, the arrest coefficient for CD8+ T cells in the PyXNL-infected mice after transfer was 77% (**Fig.** **7B**) compared to 33% in the Pyfabb/f- immunized donor mice before transfer (**Fig.** **3B**). Thus, although slightly more motile than CD8+ T cells from Py-RAS immunized mice, CD8+ T cells from Py-fabb/f(−) mice also remained locally confined [76]. IVM as well as *ex vivo* examination of the entire liver surface provided no evidence for LS development 18 h post infection in the recipient mice. Others used PCR to show that 20–40 million cloned CD8+ T cells strongly inhibited the development of 100 *P. yoelii* sporozoites in the liver, even when adoptively transferred as late as 20 h after infection [80], [81]. In another study, inoculation of 6 million activated *P. yoelii* CSP tetramer-positive CD8+ T cells resulted in a near-complete inhibition of the development to LS of 50,000 simultaneously inoculated sporozoites [82]. It should be noted that in our efforts to observe interactions between CD8+ T cells and LS, we inoculated sporozoite numbers that exceed those typically used to confirm protection after challenge by several logs of magnitude. Thus, while we expect the adoptively transferred CD8+ T cells to have reduced the parasite biomass in the liver, some LS likely survived under these experimental conditions.

Sterile protection of BALB/c mice by Py-RAS immunization requires the induction of a large percentage of parasite-specific CD8+ T cells [15], [59]. To increase the likelihood of detecting Py-specific CD8+ T cells within the overall pool of adoptively transferred immune cells in our IVM studies, we utilized a heterologous prime/boost immunization protocol that has been shown to generate high levels of CD8+ memory T cells specific for the Py CTL epitope CS_280–288_ [66], [67]. In this protocol, BALB/c mice were primed with 1×10^6^ splenic dendritic cells coated with *P. yoelii* CS_280–288_ and boosted 7 d later with 1×10^7^ CFU recombinant *Listeria monocytogenes* expressing CS_280–288,_ as described previously [66], [67]. Another group of BALB/c mice was then infected with PyXNL-GFP, as above, followed by immediate inoculation of 10^6^ purified splenic CD8+ T cells from the Py-CS_280–288_ immunized mice. Py-CS_280–288_ specific TCR transgenic cells represented 6.2% and 28.2% of the total and CD8-gated splenocytes, respectively (**Fig. S2**). Similar to the CD8+ T cells from Py-RAS or Pyfabb/f(−) immunized mice, the CD8+ T cells from the Py-CS_280–288_ immunized mice exhibited a low average velocity (1.06±0.26 μm/min) and a high arrest coefficient (89%) at 18 h post infection compared to CD8+ T cells in immunized donor mice before transfer (**Fig.** **7** **A and 7B** ).

## Discussion

The major finding of this study is that adoptive transfer renders CD8+ T cells immobile in the sinusoidal microvasculature for a period of at least 3 days. While motile and actively patrolling the liver of immunized donor mice, few of the adoptively transferred CD8+ T cells exhibited significant motility, neither in infected nor in uninfected mice. This loss of motility was observed for CD8+ T cells from donor mice immunized with Py-RAS, Pyuis4(−), or Pyfabb/f(−) sporozoites, as well as for Py-CS_280–288_ epitope-specific CD8+ T cells. In this study, adoptive transfer was done under conditions known to confer protection against challenge with a small number of viable sporozoites [20]. To allow monitoring of multiple LS per IVM session, mice were infected with 2–3 logs higher sporozoite numbers than what has been typically used for challenge. Although the resulting higher LS density should have facilitated parasite recognition, surprisingly none of the adoptively transferred CD8+ T cells made contact with infected hepatocytes under any of the experimental conditions used. While motile CD8+ T cells should be able to use the entire arsenal of contact-dependent and -independent cytotoxic mechanisms to eliminate *Plasmodium* LS from the liver, we speculate that the initially immobile adoptively transferred CD8+ T cells have only soluble mediators at their disposal and use cytokines, in particular IFN-γ, for parasite killing. A central role of IFN-γ in protection against *P. yoelii* was demonstrated using a DC prime/*L. monocytogenes* boost regimen, a model in which CD8+ memory T cells represent the only *Plasmodium*-specific immune cells [66]. We further speculate that adoptive transfer of CD8+ T cells from granzyme or perforin deficient mice immunized with this prime boost regimen would prevent liver infection to a similar degree as transfer of CD8+ T cells from immunized wild type mice.

### CD8+ T cells use redundant effector mechanisms for *Plasmodium* LS elimination

It has been known for several decades that immunization with radiation-attenuated sporozoites stimulates a strong protective response [6], [7], [83]. However, the cellular interactions involved in the elimination of *Plasmodium* LS from the liver *in vivo* are still unknown. CD8+ T cells are instrumental in protection and it is currently assumed that effector T cells use redundant mechanisms of cytotoxicity that operate in parallel [53], [59], [66]. While CD8+ T cells are clearly capable of parasite killing via immunological synapse formation in hepatocyte monocultures *in vitro* [56], [58], the situation in the natural hepatic microenvironment, i.e. in the presence of non-parenchymal cells, blood flow, and a normal liver architecture, is more complex [51], [59]. Studies using a variety of different *in vivo* models, including knockout mice, antibody-mediated ablation, synthetic cytokines and adoptive T cell transfer, suggest that direct CD8+ T cell-mediated cytotoxicity, involving perforin, granzyme B, Fas, FasL, or TRAIL represents the predominant mechanism of protection [51], [58], [66], [84], [85] and that immunity can be achieved in the absence of IFN-γ or IFN-γ signaling [51], [66], [73], [80], [86]–[89]. On the other hand, a role of IFN-γ and TNF-α in parasite killing is clearly documented, both *in vivo* and *in vitro* [25], [51], [66], [81], [85], [90]–[96]. Although the IFN-γ mediated iNOS upregulation in infected hepatocytes is considered crucial for protection [92], [97], [98], the exact mode of operation of this cytokine *in vivo* is not established (reviewed in [51], [53], [66]).

Despite the utility of adoptive transfer experiments for the study of CD8+ T cell function in response to vaccination against malaria, this approach remains sparsely used because inordinate numbers of CTLs are required to effectively eliminate liver stage malaria parasites. Our finding that adoptive transfer renders CD8+ T cells immobile and thus unable to form an immunological synapse with infected hepatocytes for a period of at least 3 days may explain this and also shed light on some of the apparent contradictions in the literature. In agreement with the notion that CD8+ T cells are initially immobile and thus unable to use contact-dependent mechanisms of cytotoxicity to attack and eliminate *Plasmodium* LS from the liver, antibody-mediated blockage of IFN-γ abrogated protection within 2 days [81], [93], [99]. Protection was not abolished, however, when IFN-γ was neutralized in actively immunized mice [51] or when mice were challenged 8 days after transfer of CD8+ T cells from IFN-γ deficient mice [73]. Together, these and our data suggest that CD8+ T cell mobility and a complete repertoire of cytotoxic mechanisms are regained within a week after adoptive transfer. This notion is supported by the finding that CD8+ T cells from IFN-γ KO mice protect recipient mice 8 days after adoptive transfer [73] and that CD8+ T cells are able to protect IFN-γ KO mice against infection with *P. yoelii*, presumably by using a contact-dependent cytotoxic mechanism [66]. Based on these reports and our IVM observations, we propose a model in which CD8+ T cells actively monitor the liver sinusoids of immunized mice and eliminate LS via a combination of redundant mechanisms that include classical granule-mediated cytotoxicity [51], [66] as well as the cytokines IFN-γ and TNF-α [66], [94], [98], [100], with additional IFN-γ provided by hepatic NK cells through a positive feedback loop that involves Kupffer cell or DC-derived IL-12 [101]–[103]. Thus, adoptive transfer induced T cell immobilization could be used as a model to elucidate the mode of action of cytokines against infected hepatocytes in the absence granule-mediated T cell cytotoxicity. Further analysis of the response of resident intrahepatic CD8+ T cells of immunized mice to challenge with viable sporozoites and of adoptively transferred CD8+ T cells to immunization with attenuated parasites should reveal how accurately the adoptive transfer model represents the events that occur during a natural challenge.

### Lymphatic fluid transport in the liver

The liver is widely recognized as a lymphatic organ with unique immunological properties [43], [45], [46], [104]–[106], but its role in lymph formation and the resulting implications for liver immunology have been largely discounted to date. According to the current model, plasma continuously enters the space of Disse through the sinusoidal sieve plates, and flows as lymph retrogradely around the sinusoids towards the periportal space of Mall [107]–[110]. This blood/lymph counterflow concept has important implications for hepatic immunology (reviewed in [61]). We speculate that the perisinusoidal lymphatic counterflow would transport secreted cytokines along the space of Disse towards the portal field thus directly exposing infected hepatocytes to these cytotoxic mediators, if *Plasmodium*-specific effector T cells were to eliminate LS via cytokines such as IFN-γ from within the sinusoidal lumen. The potential use of soluble mediators for CD8+ T cell mediated *Plasmodium* LS killing is reminiscent of data from a murine *Leishmania* model, in which CD4+ T cells were shown to exert their protective activity by generating a gradient of IFN-γ that reaches more than 80 μm beyond the site of antigen presentation thus engaging a minority of infected cells and promoting pathogen clearance in the absence of immunological synapse formation [60]. Unlike in the skin, however, where interstitial fluid diffuses slowly through the intercellular spaces, the liver parenchyma is highly vascularized, both in terms of blood and lymph vessels, and contains, except for the portal fields, very little connective tissue. Cytokine dissemination in the liver must therefore be expected to occur considerably faster than in the skin. Considering the high sinusoid-to-lymph filtration rate [110]–[112], the enhanced pressure gradients created by leukocytes moving through the sinusoidal lumen [113], and the highly anastomozed nature of the sinusoidal microvasculature, we speculate that immobilized CD8+ T cells could conceivably exploit both the anterograde blood flow in the sinusoids and the retrograde lymph flow in the perisinusoidal spaces of Disse to take control of a substantial portion of the liver lobule (see [61] for a recent review).

### Implications for cross-protection

As adoptively transferred CD8+ effector T cells confer protection under the experimental conditions used here, but cannot detect antigen presented on infected hepatocytes, they could potentially kill LS in a species-independent manner. Accordingly, immunization with *P. falciparum* sporozoites or attenuated *P. berghei* or *P. yoelii* sporozoites resulted in cross-protection against heterologous challenge [18], [23], [26], [114]–[118]. Although cross-protection appears to depend on a blood factor [117], several experimental systems have shown that antibodies are not involved [91], [114], [119] (reviewed in [115]). Comparison of the various attenuation and immunization strategies used over the past years suggests that the superior cross-protective immunity elicited by genetically modified late-stage arresting parasites may be due to improved IFN-γ production [18]. Together with the motility data presented here, we speculate that CD8+ T cells are able to recognize parasite antigens on non-parenchymal cells, in particular Kupffer cells [47], [120], and kill LS, including those located at somewhat larger distances, in a species-independent fashion by secretion of large amounts of IFN-γ into the sinusoidal microvasculature. Interestingly, proinflammatory cytokine mediated cross-protective mechanisms have also been reported to operate against malaria blood stages as well as other intracellular blood-borne pathogens (reviewed in [121]).

### LS development in immunized mice

Under natural exposure conditions, effector T cells are unlikely to encounter mature *Plasmodium* LS. First, most sporozoites are immobilized by antibodies after transmission by mosquito bite and thus unable to exit the skin [30]. The few parasites that do reach the liver are likely opsonized and phagocytozed by Kupffer cells [122], [123]. Should individual sporozoites succeed in infecting hepatocytes, *Plasmodium*-specific liver resident CD8+ effector memory T cells would likely kill the growing LS before they reach maturity [78], [124]. In agreement with this notion, sporozoite infection of immunized mice resulted in a drastic reduction in LS number compared to naïve mice (**Fig.** **1**). Surprisingly, we also found that the few surviving LS were of a significantly smaller size. Neither Hoechst nor MitoTracker staining provided any evidence for chromatin condensation or mitochondrial damage suggesting that parasite growth retardation was not due to host cell death. Further, DNA staining revealed that the small LS formed merozoites at very late stages of development, which would not have been the case if host cell or parasite death had been the reason for the small parasite size. Examination of the blood of these mice by Giemsa staining and wet mounts starting 2 days after challenge revealed a few fluorescent merozoites, but no detectable increase in parasitemia over the course of 2 weeks. For these reasons, we speculate that other factors, for example cytotoxic effects exerted by proinflammatory cytokines or parasite-specific antibodies some of which are reportedly able to inhibit the intracellular LS development [125], [126], were responsible for the observed reduction in growth rate. While inflammatory cytokines such as IFN-γ are generally thought to eliminate LS via the NO pathway, an intriguing alternative may be that severely growth-inhibited, and thus barely detectable, miniscule parasites persist in the liver of immune mice and contribute to the formation of the depot of LS antigen that is required for maintenance of protracted protective immunity [78].

### Possible causes for CD8+ T cell immobilization

There are several scenarios we considered that could explain the observed reduced CD8+ T cell motility after adoptive transfer. First, it could be argued that the hepatic environment of naïve mice differs substantially from that of immunized mice. A general change in the immunized animal could potentially explain why CD8+ T cells appear to regain the ability to kill via classical contact-dependent cytotoxicity within 8 days after transfer. This hypothesis could be tested by measuring the velocity of CD8+ T cells transferred from one into another immunized mouse. Second, only a small number of antigen specific T cells were transferred and thus utilized under our experimental conditions. However, the lack of motility of the Py-CS_280–288_ specific CD8+ T cells in the time frame considered and the fact that CD8+ T cells of both hepatic or splenic origin equally showed reduced motility, despite the liver harboring considerably more parasite-specific CD8+ effector memory T cells than the spleen [22], [25], [127], would argue against this possibility. Third, the transfer protocol could be the culprit, and we consider this the most likely explanation. Adoptive transfer rendered the entire population of splenic CD8+ T cells immobile in the liver, suggesting that T cell immobility after transfer was antigen-independent, and therefore likely not recoverable by another immunization. We favor a model in which CD8+ T cells undergo a general phenotypic change during the adoptive transfer procedure. For example, the activation status of the memory T cells could have been affected after removal from the donor mice. Alternatively, *de novo* expression of adhesion molecules on the surface of CD8+ T cells could have been stimulated. In agreement with the higher expression levels of the integrins ICAM-1 and VCAM-1 in the liver compared to other tissues [128], activated CD8+ T cells were shown to be trapped in the liver in two ways, by an active antigen-dependent mechanism based on ICAM-1/LFA-1 and a passive antigen-independent mechanism based on VCAM-1/VLA-4 [129]. According to the current model, activated CD8+ T cells are initially sequestered in the liver by passive adhesion. Antigen recognition on the surface of hepatocytes or endothelia then increases the affinity of the interaction, mainly with ICAM-1, thus extending T cell residence time in the liver [129]. It is therefore conceivable that the transferred CD8+ T cells upregulated LFA-1 in response to the purification and adoptive transfer procedures and arrested in the liver by binding to ICAM-1. Of note, ICAM-1 is constitutively expressed on both sinusoidal endothelia and Kupffer cells [128]. Alternatively, high levels of the adhesion molecule CD44 is considered the most reliable surface marker of CD8+ T cell activation and has been implicated in effector memory CD8+ T cell cytotoxicity against *Plasmodium* LS [74], [124], [130]–[132]. CD44 is involved in the sequestration of neutrophils in the liver [133] suggesting that this ligand of hyaluronic acid could also contribute to the observed CD8+ T cell immobilization in the liver. Insight into these molecular events, in combination with direct intravital observations, will provide critical information on the value of adoptive transfer techniques for analysis of the mechanisms leading to CD8+ T cell mediated elimination of *Plasmodium* LS, and perhaps other intracellular pathogens from the liver.

## Conclusions

Based on our findings and published evidence, we propose a model in which CD8+ T cells can recognize antigen presented by non-parenchymal hepatic APC, in particular Kupffer cells, and take advantage of the unique fluid transport pathways of the liver, namely the anterograde bloodstream and the retrograde lymphatic flow, for efficient dissemination of pro-inflammatory mediators towards infected hepatocytes. The ensuing NO formation then allows destruction of the intracellular parasites. Cytokine-enhanced cell-mediated cytotoxicity could potentially remove the necessity for effector T cells to screen every single hepatocyte to eliminate a minute number of *Plasmodium* LS from a huge organ such as the liver. In combination with classical granule-mediated cytotoxicity, this contact-independent mechanism would not only dramatically increase the efficiency of finding the infamous needle in the haystack, but should also provide cross-protection against other *Plasmodium* species. A better understanding of these effector functions will guide the rational design of future malaria vaccines and adjuvant formulations that stimulate immune responses comparable to or better than those observed during parasite infection to interrupt the clinically silent liver phase of the *Plasmodium* life cycle.

## Supporting Information

Figure S1 **Velocities of IHL and splenic CD8+ T cells in PyXNL-GFP infected mice.** IHL (Liver) or splenic (Spleen) CD8+ T cells were purified from immunized mice 2 weeks after the second booster with Py-RAS and adoptively transferred into PyXNL-GFP infected recipient mice. Velocities were measured at 18 h or 40 h post infection. At least four infected mice were used per experimental condition. NS  =  not significant (p>0.05).
(TIF)

Figure S2 **Frequency of CS_280–288_ specific TCR-transgenic CD8+ T cells.** Naive BALB/c mice (Thy1.2) were seeded with 2000 naive Thy1.1 CS_280–288_-specific TCR-transgenic cells. Recipient mice were primed with 10^6^ mature dendritic cells coated with the CS_280–288_ peptide and boosted one week later with 5×10^6^ attenuated recombinant *L. monocytogenes* expressing the CS_280–288_ epitope. Splenic memory cells were evaluated >60 days after boosting. Total splenocytes (left) or CD8 gated splenocytes (right) were stained with isotope controls (top) or anti-CD8 and anti-Thy1.1 (bottom) to determine the frequency of CS_280–288_-specific TCR-transgenic cells.
(TIF)

Video S1 **Behavior of CD8+ T cells in a naive Tie2-GFP liver.** Recording of multiple Z-stacks over time (XYZT) showing a naive mouse with GFP+ endothelia (green), anti-CD8a-PE labeled CD8+ T cells (green), MitoTracker labeled hepatocyte mitochondria (red), and Hoechst stained nuclei (blue). See Figure 2A for representative still image. Scale bars 20 μm.
(AVI)

Video S2 **Behavior of CD8+ T cells in a Pyfabb/f(−) immunized mouse liver.** Recording of multiple Z-stacks over time (XYZT) showing a Pyfabb/b- immunized mouse liver with anti-CD8a-PE labeled CD8+ T cells (green), MitoTracker labeled hepatocyte mitochondria (red), and Hoechst stained nuclei (blue). See Figure 2B for representative still image. Scale bars 20 μm.
(AVI)

Video S3 **Behavior of CD8+ T cells in a Py-RAS immunized mouse liver.** Recording of multiple Z-stacks over time (XYZT) showing a Py-RAS immunized mouse liver with anti-CD8a-PE labeled CD8+ T cells (green), MitoTracker labeled hepatocyte mitochondria (red), and Hoechst stained nuclei (blue). See Figure 2C for representative still image. Scale bars 20 μm.
(AVI)

Video S4 **Behavior of CD8+ T cells in a Pyuis4(−) immunized mouse liver.** Time sequence (XYT) showing a Pyuis4(**−**) immunized mouse liver with anti-CD8a-PE labeled CD8+ T cells (red) and MitoTracker labeled hepatocyte mitochondria (green). See Figure 2D for representative still image. Scale bars 20 μm.
(AVI)

Video S5 **Behavior of Py-RAS activated CD8+ T cells in a PyXNL-GFP infected mouse liver**. Recording of multiple Z-stacks over time (XYZT) showing an 18 h PyXNL-GFP LS (bright green) with a nearby Calcein Red Orange labeled CD8+ T cell (red). Hoechst labeled nuclei are blue, the tissue was visualized by collecting autofluorescence (green). See Figure 4A for representative still image. Scale bars 20 μm.
(AVI)

Video S6 **Behavior of Py-RAS activated CD8+ T cells in a PyXNL-GFP infected mouse liver.** Recording of multiple Z-stacks over time (XYZT) showing a PyXNL-GFP LS (green) and several CD8+ T cells labeled with CellTrace Calcein Violet (blue) in the sinusoidal space at 42 h post infection. Hepatocyte mitochondria were visualized with MitoTracker Deep Red (red). See Figure 4B for representative still image. Scale bars 20 μm.
(AVI)

Video S7 **Behavior of Py-RAS activated CD8+ T cells in a PyXNL-GFP infected mouse liver**. Recording of multiple z-stacks over time (XYZT) showing a Calcein Red Orange labeled CD8+ T cells (blue) in the sinusoidal space at 18 h post infection. Hepatocyte mitochondria were visualized using MitoTracker Green (green). Scale bars 20 μm.
(AVI)
