# Supplementary figures and images for: In vivo CD8+ T Cell Dynamics in the Liver of Plasmodium yoelii Immunized and Infected Mice
Source: PLoS One. 2013 Aug 14;8(8):e70842. doi: 10.1371/journal.pone.0070842 (PMC3743839; doi:10.1371/journal.pone.0070842)

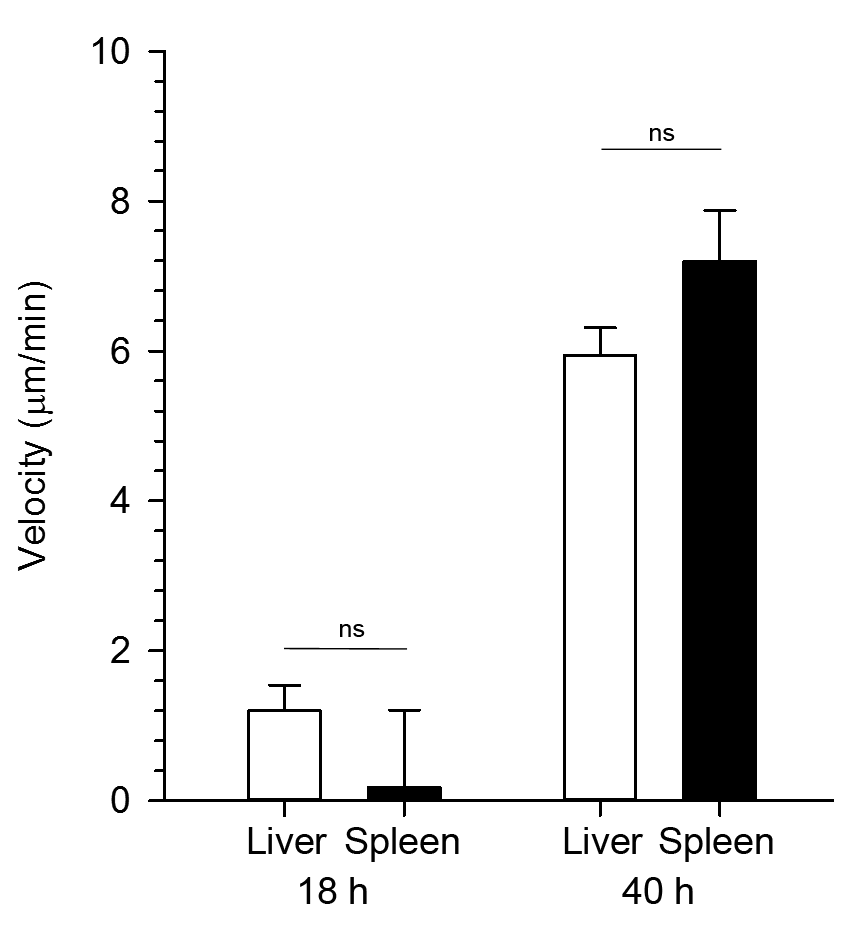

Supplement: Figure S1 — Velocities of IHL and splenic CD8+ T cells in PyXNL-GFP infected mice. IHL (Liver) or splenic (Spleen) CD8+ T cells were purified from immunized mice 2 weeks after the second booster with Py-RAS and adoptively transferred into PyXNL-GFP infected recipient mice. Velocities were measured at 18 h or 40 h post infection. At least four infected mice were used per experimental condition. NS = not significant (p>0.05). (TIF) [file pone.0070842.s001.tif]

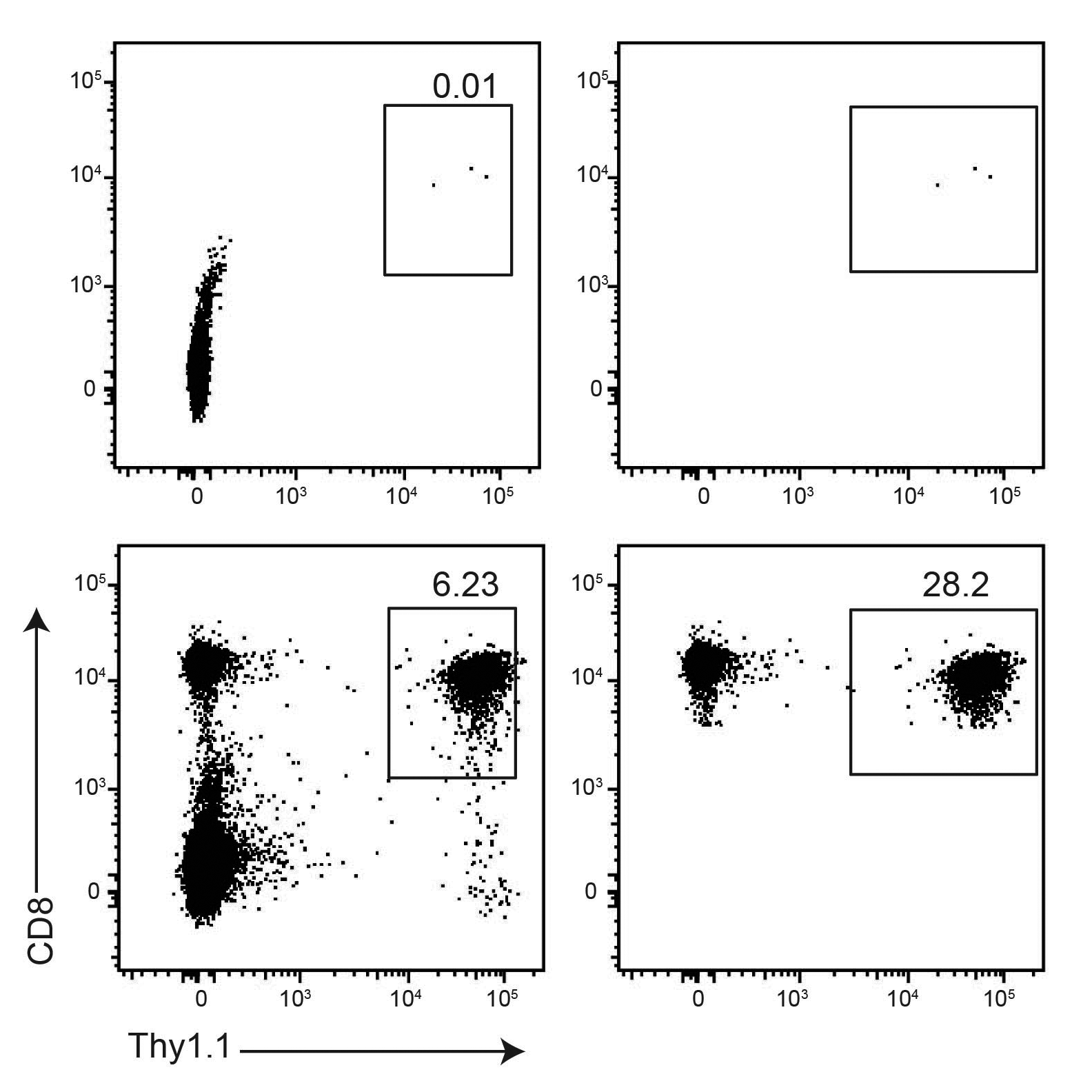

Supplement: Figure S2 — Frequency of CS280–288 specific TCR-transgenic CD8+ T cells. Naive BALB/c mice (Thy1.2) were seeded with 2000 naive Thy1.1 CS280–288-specific TCR-transgenic cells. Recipient mice were primed with 106 mature dendritic cells coated with the CS280–288 peptide and boosted one week later with 5×10^6^ attenuated recombinant L. monocytogenes expressing the CS280–288 epitope. Splenic memory cells were evaluated >60 days after boosting. Total splenocytes (left) or CD8 gated splenocytes (right) were stained with isotope controls (top) or anti-CD8 and anti-Thy1.1 (bottom) to determine the frequency of CS280–288-specific TCR-transgenic cells. (TIF) [file pone.0070842.s002.tif]
